# Epiregulin increases stemness-associated genes expression and promotes chemoresistance of non-small cell lung cancer via ERK signaling

**DOI:** 10.1186/s13287-022-02859-3

**Published:** 2022-05-12

**Authors:** Yujia Zhang, Fengjun Qiu, Tingjie Ye, Sau Har Lee, Jiatuo Xu, Lingyan Jia, Rui Zeng, Xiaoling Wang, Xudong Hu, Xiaofeng Yan, Hua Li, Yanlin Lu, Xiaoling Wang, Rilei Jiang, Wei Xu

**Affiliations:** 1 School of Basic Medical Science, Shanghai University of Traditional Chinese Medicine, Shanghai, 201203 China; 2 School of Biosciences, Faculty of Health and Medical Sciences, Taylor’s University, Lakeside Campus, Subang Jaya, Selangor Malaysia; 3 ZJU-UoE Institute, Zhejiang University School of Medicine, Zhejiang University, Haining, China; 4 Department of Oncology and Institute of Traditional Chinese Medicine in Oncology, Longhua Hospital, Shanghai University of Traditional Chinese Medicine, Shanghai, China

**Keywords:** Non-small cell lung cancer, Chemoresistance, Receptor tyrosine kinase, EREG, Stemness, ERK signaling

## Abstract

**Background:**

Chemoresistance often causes the failure of treatment and death of patients with advanced non-small-cell lung cancer. However, there is still no resistance genes signature and available enriched signaling derived from a comprehensive RNA-Seq data analysis of lung cancer patients that could act as a therapeutic target to re-sensitize the acquired resistant cancer cells to chemo-drugs. Hence, in this study, we aimed to identify the resistance signature for clinical lung cancer patients and explore the regulatory mechanism.

**Method:**

Analysis of RNA-Seq data from clinical lung cancer patients was conducted in R studio to identify the resistance signature. The resistance signature was validated by survival time of lung cancer patients and qPCR in chemo-resistant cells. Cytokine application, small-interfering RNA and pharmacological inhibition approaches were applied to characterize the function and molecular mechanism of EREG and downstream signaling in chemoresistance regulation via stemness.

**Results:**

The RTK and vitamin D signaling were enriched among resistance genes, where 6 genes were validated as resistance signature and associated with poor survival in patients. EREG/ERK signaling was activated by chemo-drugs in NSCLC cells. EREG protein promoted the NSCLC resistance to chemo-drugs by increasing stemness genes expression. Additionally, inhibition of EREG/ErbB had downregulated ERK signaling, resulting in decreased expression of stemness-associated genes and subsequently re-sensitized the resistant NSCLC cells and spheres to chemo-drugs.

**Conclusions:**

These findings revealed 6 resistance genes signature and proved that EREG/ErbB regulated the stemness to maintain chemoresistance of NSCLC via ERK signaling. Therefore, targeting EREG/ErbB might significantly and effectively resolve the chemoresistance issue.

**Supplementary Information:**

The online version contains supplementary material available at 10.1186/s13287-022-02859-3.

## Introduction

Lung cancer ranks first in terms of mortality worldwide, especially in China [1]. NSCLC is the most predominant pathological subtype of lung cancer, accounting for approximately 85% in all cases [2, 3]. National Institute for Health and Care Excellence (NICE) recommended docetaxel plus platinum or platinum-doublet for the treatment of patients with locally advanced or metastatic NSCLC. However, these chemo-drugs treatment often caused resistance eventually, as evidenced in multiple studies that have reported tumor recurrence or drug resistance in approximately 70% of the treated patients [4, 5]. Resistance is often a leading cause of therapeutic failure, hence limiting the clinical application of chemo-drugs in patients with advanced NSCLC [6]. Therefore, it is important to identify the resistant cancer cells population and to subsequently elucidate the resistance mechanism in NSCLC.

There have been many studies that reported the resistance signature in gastric cancer [7], colorectal cancer [8], glioma [9], pancreatic cancer [10] and even lung cancer [11]. A previous study in lung cancer has revealed a 35-gene resistance signature and has identified JmjC KDMs inhibitor, JIB-04, as a promising drug for targeting taxane-platin-chemoresistant NSCLCs; however, this study utilized NSCLC cell lines as their study model to develop the expression profiles, rather than investigating primary lung cancer specimens to enrich the resistance signature [11]. On top of that, the researcher had only identified a new target for killing the resistant NSCLC cells without any suggestion on how to reverse the resistance ability while increasing the sensitivity of resistant lung cancers to chemo-drugs.

The ErbB family of receptor tyrosine kinase (RTK) includes EGFR, ERBB2, ERBB3 and ERBB4, which were frequently associated with malignant proliferation of tumor cells [12]. These receptors were bound by variants of cytokines and activated to induce intracellular signaling. It was demonstrated that EGFR overexpression had rendered breast cancer resistant to various anticancer drugs [13]. It was also reported that the attenuation of EGFR signaling in NSCLC enhanced cisplatin sensitivity, which implies that the EGFR signaling is involved in the chemo-drug resistance [14]. Epiregulin (EREG) is a member of the epidermal growth factor family that has a similar function with EGF, which binds to the ErbB receptors to regulate the proliferation and anti-apoptosis of cancer cells [15, 16]. In the tumor tissues of NSCLC patients, 64.7% of tumors were EREG positive as shown by IHC staining. Further study also revealed that the prognosis of EREG positive patients was worse than EREG negative patients [17], implying that EREG may be related to resistance. This speculation was supported by findings from another study that showed colon cancer resistance to the 5-FU drug due to upregulation of EREG. Furthermore, subsequent inhibition of EREG had reversed the resistance of cancer cells in colon cancer [18]. Nevertheless, the regulatory function and mechanism of chemoresistance in NSCLC by EREG remains unclear.

Taken together, we aim to screen the resistance signature of NSCLC and enrich the activated signaling involved in resistance. In this study, we successfully enriched and validated the resistant genes signature through comprehensive profiling of the lung cancer patients’ data retrieved from the cancer genome atlas (TCGA) database. EREG was shown to increase stemness-associated genes expression and promoted cancer stem cells’ resistance to chemo-drugs via ERK signaling. It is concluded that enriched EREG/ErbB signaling is activated and could be a potential target for resistant lung cancer. This was demonstrated when afatinib that targeted ErbB reversed the phenotype of cancer cells from resistant to sensitive at a low concentration, which was subsequently eliminated by chemo-drugs. These findings prove our hypothesis that switching the resistant status to sensitive status by targeting resistant genes or signaling pathways is a more effective approach to resolve resistance issues in combination with the conventional chemo-drugs, as compared to drugs alteration strategy that is frequently applied in clinical treatment.

## Materials and methods

### TCGA data download and analysis

RNA-seq analysis was carried out as described by another study [19]. The RNA-Seq data of lung cancer was searched in the GDC data portal available in the TCGA database. The downloaded RNA-Seq data were processed using R studio and the differential genes were analyzed by DESeq2 package. Cutoff settings of the differential genes were *p* < 0.05 with fold change > 2. Resistant genes were screened in the treated patients compared to the non-treated patients after excluding the “not reported” cases. GO analysis of the 32 resistant genes was conducted using DAVID software.

### Survival curve analysis and meta-analysis

The survival curves of genes were plotted by online software using Kaplan–Meier plotter and gene expression profiling interactive analysis. HRs (hazard ratios), *p*(HRs) and 95% confidence intervals (CIs) were extracted for meta-analysis. The HRs and 95% CIs were applied to calculate the pooled HRs and 95% CIs. If the 95% CI of any gene was absent, it was calculated based on HRs and *p-value* using Review Manager Software, version 5.4.1.

Meta-analysis was performed using the STATA software, version 16.0 (STATA Corporation, College Station, TX, USA). Pooled HRs with 95% CIs were calculated for positive gene signatures in different cancer types and were presented in a forest plot. An HR > 1 implied that the patients who highly expressed those genes had a shorter survival time. A *p-value* less than 0.05 was considered statistically significant. Heterogeneity was calculated and presented as *I*^*2*^. The random effect model was chosen for analysis based on the fact that the studies were different in terms of sample size and patients. The detailed information of meta-analysis is available in the study published by Xu et al. [20].

### Drug preparation and storage

Cisplatin (MCE, Shanghai, China) was dissolved in RPMI-1640 medium and stored at 4 °C for future use, as described by another study [21], whereas taxol (MCE, Shanghai, China), afatinib (Selleck, Shanghai, China) and selumetinib (Selleck) were dissolved in DMSO. EREG (MCE, Shanghai, China) protein was dissolved in H_2_O. All these drugs were aliquoted and stored at − 20 °C for long term use.

### Cell culture

Cell culture was carried out as described by Fathi and Vietor with some modifications [22]. A549 and H1299 cells were cultured in RPMI-1640 medium (Gibco, MA, USA) with 10% fetal bovine serum (GeminiBio, CA, USA). A549 cells were treated with gradual increment of 1 μg/mL, 2 μg/mL and 4 μg/mL cisplatin for 4–5 days before this established cisplatin resistant cells were validated by CCK-8 assay. Similarly, A549 cells were treated with gradual increment of 30 ng/mL, 60 ng/mL and 100 ng/mL taxol for 4–5 days followed by validation of these established taxol-resistant cell by CCK-8 kit (Bimade, Shanghai, China). These cells were treated with inhibitors combined with chemo-drugs and harvested for RNA and protein extraction. All human cell lines have been authenticated using STR profiling within the last three years and all experiments were performed with mycoplasma-free cells.

### Establishment of shRNA knocked down cell

This experiment was conducted as described by He et al. [23]. The EREG shRNA lentivirus was purchased from genepharma company (Shanghai, China) with the target sequence as *shEREG1* (GCTCTGACATGAATGGCTATT) and *shEREG2* (GCATGGACAGTGCATCTATCT). The cells were digested by 0.25% trypsin (Gibco, USA) and resuspended to 2–5 × 10^4^ /mL. Two milliliter resuspended cells were added into 6 well plates. After incubation overnight, 10 μL lentivirus and 2 μL polybrene were added into the wells. 2 μg/mL Puromycin was added into the wells for selection of the infected cells after 2–3 days.

### Quantitative RT-PCR

Quantitative RT-PCR analysis was performed in accordance to the protocol described by Adibkia et al. [24]. Total ribonucleic acid (RNA) was extracted using Trizol reagent (Invitrogen, MA, USA) and was reverse-transcribed into cDNAs using the StarScript II first-strand cDNA synthesis kit (Yeason, Shanghai, China), according to the manufacturers’ instructions. The cDNAs were amplified by quantitative RT-PCR using the Universal SYBR Green mix (Bimake). GAPDH was used as an internal reference to normalize the input cDNAs. All the RT-PCR primer sequences used in this study are listed in Additional file 10: Table S5.

### Western blot

This experiment was conducted as described by Fathi et al. with some modifications [25]. Total proteins were extracted with a lysis buffer consisting of 150 mM NaCl (Merck, NJ, USA), 1.0% Triton X-100, 1% sodium deoxycholate (Merck), 0.1% SDS (Amresco) and 50 mM Tris–Cl pH 8.0 (Merck), followed by centrifugation at 14,000 rpm for 10 min at 4 °C. Extracted proteins (20 μg) were denatured and resolved in 10% SDS-PAGE gels. The separated protein bands were then transferred onto PVDF membrane. These blots were incubated with the primary antibodies at 4 °C overnight, followed by incubation with secondary antibodies at room temperature for 1 h. After subsequent incubation with ECL solution (Genenorth, Beijing, China), chemiluminescence signal on the blots were captured using the ChemiDox XRS + system (Bio-Rad, Hercules, CA). The primary antibodies used in this study are listed in Additional file 11: Table S6.

### Cell viability assay

The cells were digested by 0.25% trypsin and resuspended to 2 ~ 5 × 10^4^/mL. 100 μL resuspended cells were then seeded into 96 well plates. After overnight incubation, the wells were replaced with medium containing drugs and kept for another 48 h. Detection of cells viability was done as described by Wu et al. [26]. 10 μL of CCK-8 (Bimake) reagent was added into each well and maintained for 2–4 h. The absorbance was detected with a plate reader at a wavelength of 450 nM.

### Sphere forming assay

The sphere formation assay was conducted in accordance to Zhao et al. [27]. The cells were digested by 0.25% trypsin and resuspended in 1 × PBS. Cell density was adjusted to 1 × 10^4^/mL. 20 μL cells suspension was added into 500 μL sphere forming medium containing DMEM-F12 (Gibco), 1 × B27 (Absin, Shanghai, China), 10 ng/mL EGF (Absin), and 10 ng/mL bFGF (Absin). These cells were cultured at 37 °C in an incubator for 4–7 days. Spheres were imaged under a microscope and positive spheres larger than 50 μm in diameter were counted using image-J software.

### Statistical analysis

Data were reported as means ± SEM of at least three replicates. Mean differences were compared using two-sided Student’s t-tests. *P* value lesser than 0.05 was considered to be statistically significant. Error bars, mean ± SEM; n.s., *p* > 0.05; *, *p* < 0.05; **, *p* < 0.01.

## Results

### Differential genes in lung cancer

**Fig. 1:**
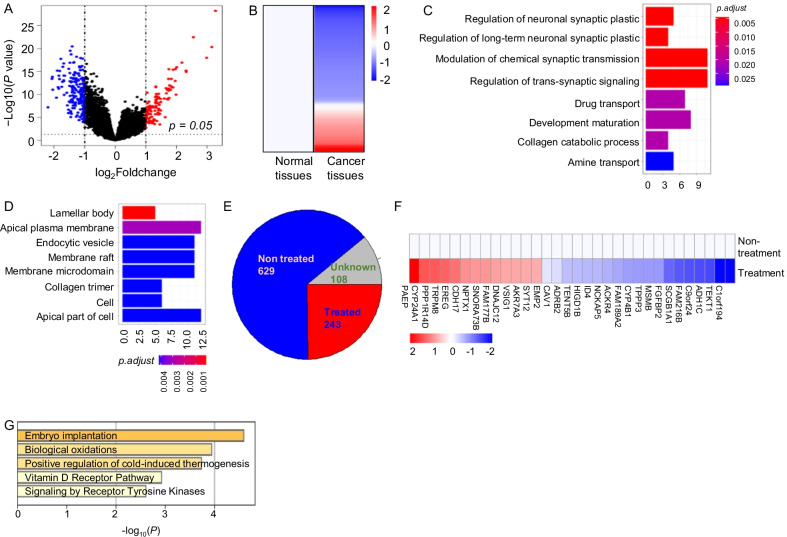
The differential genes and resistance-associated genes in lung cancers tissues vs normal lung tissues. **A** The Volcano plot for the differential genes at twofold-change in 998 lung cancer tissues compared with 103 normal lung tissues from TCGA database (the blue dot, 189 downregulated genes; the red dot, 105 upregulated genes; *p* < 0.05). **B** The heatmap for the 294 differential expression genes at twofold-changes. **C** The enriched biology process for the 105 upregulated genes. **D** The enriched cell components for 189 downregulated genes. **E** The pie chart for classification of the 998 lung cancer patients. **F** The heatmap for 32 resistant genes in non-treated and treated patients. **G** The GO analysis of 32 resistant genes

RNA-Seq data from 103 normal lung tissues and 998 lung cancer tissues were analyzed. The differentially expressed genes included 189 downregulated genes and 105 upregulated genes at twofold changes level as shown in volcano map and heatmap (Fig. 1A and B). The 105 upregulated genes were further analyzed in enriched biological process and it had showed drug transport and collagen catabolic processes were enriched in these upregulated genes (Fig. 1C and Additional file 6: Table S1), which indicated that these genes were associated with the drug resistance and tumor metastasis activities. Afterward, we enriched the cellular components related to the 189 downregulated genes and it was found that these genes suppressed in lung cancers were typically associated with apical plasma membrane, collagen trimer and apical part of cell components (Fig. 1D and Additional file 7: Table S2), which therefore also indicated a possible correlation of these genes with tumor metastasis.

### Resistant genes signature in lung cancer

The enriched drug transport process among the upregulated genes implied that these genes rendered the lung cancer resistance to drugs (Fig. 1C and Additional file 6: Table S1). However, it was necessary to firstly screen the resistance genes from the patients that were under chemo-drugs therapy. To screen the resistance-related genes, we had analyzed the differential genes in line with the clinical information. According to the information, we found that there were 629 patients without treatment while 243 patients with treatment among the 998 lung cancer patients (Fig. 1E). The differential genes between non-treatment and treatment groups were analyzed and our findings revealed that there were 32 differential genes, including 13 upregulated genes with 19 downregulated genes (Fig. 1F and Additional file 1: Fig. S1). The GO analysis for these differential genes revealed that vitamin D receptor signaling and receptor tyrosine kinases signaling pathway were activated in resistant lung cancers (Fig. 1G). As shown in Additional file 8: Table S3, vitamin D receptor signaling includes *ADRB2, CYP24A1, ID4* genes, whereas receptor tyrosine kinase signaling includes *CAV1, EREG, ID4, FGFBP2, ADH1C* genes.

To prove that these genes were really correlated to resistance in lung cancer, we analyzed the hazard ratios of first progression survival time between the patients who highly expressed the genes and the patients who lowly expressed the genes. It was found that the HRs of all upregulated genes were greater than 1, which indicated that the patients who highly expressed the upregulated genes had a shorter survival time and implied these genes were positively associated with resistance (Additional file 9: Table S4).

### Resistant cells display stemness and EMT ability

**Fig. 2:**
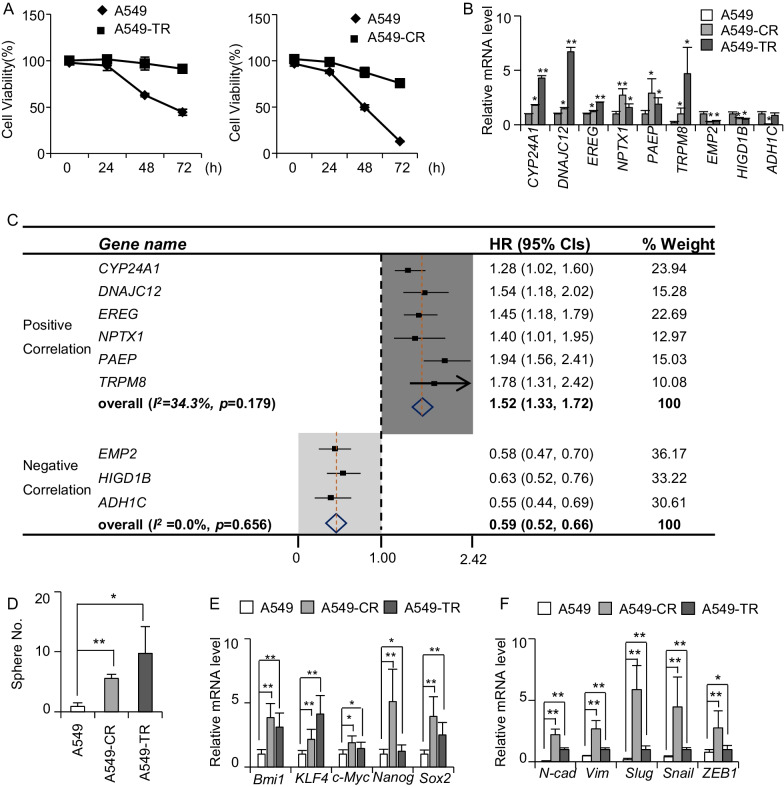
The established acquired resistant cells had stemness and EMT ability. **A** The cell viability of A549-CR or A549-TR cells treated with 4 µg/mL cisplatin or 100 ng/mL taxol for 72 h, n = 3. **B** RT-qPCR detection for the screened resistant genes in A549-CR or A549-TR cells, n = 3. **C** The hazard ratios of first progression survival time for the validated resistant genes between highly expressed population and lowly expressed population. **D** The spheres forming ability of the A549-CR or A549-TR cells compared with A549 cells (2000 cells input, n = 3). **E** RT-qPCR detection for stemness-associated genes expression A549-CR or A549-TR cells compared with A549 cells, n = 3. **F** RT-qPCR detection for EMT-related genes expression in A549-CR or A549-TR cells compared with A549 cells, n = 3. h, hours; CR, cisplatin resistance; TR, taxol resistance; HR, hazard ratio; CIs, confidence interval; *, *p* < 0.05; **, *p* < 0.01

To validate that the screened resistance genes were actually associated with resistance, we established cisplatin resistant (A549-CR) and taxol-resistant (A549-TR) A549 cells, as shown in Fig. 2A. The RT-qPCR assessment of the resistant genes in A549-CR and A549-TR cells revealed 6 positive resistance genes and 3 negative resistance genes (Fig. 2B). The positive resistance genes included *CYP24A1, DNAJC12, EREG, NPTX1, PAEP,*, and *TRPM8*. Meanwhile, the negative resistance genes included *EMP2, HIGD1B* and *ADH1C*. Meta-analysis for pooled HRs of first progression survival between lung cancer patients who highly expressed these genes and who lowly expressed these genes was conducted and it was found that the pooled HRs (95% CIs) of positive resistance genes were 1.52 (1.33, 1.72) while pooled HRs (95% CIs) of negative resistance genes were 0.59 (0.52, 0.66) (Fig. 2C). Taken together, this result demonstrated that these positive resistance genes were supposed to be the resistance gene signature in NSCLC.

Our previous study prevailed that lung cancer stem cells had resistance ability. Herein, we also wondered that whether these acquired resistant cells also possessed cancer stem cell properties. Thus, sphere forming ability of A549-CR or A549-TR cells was assessed and it was found that these drug-resistant cells showed stronger ability to form spheres as compared to A549 cells (Fig. 2D and Additional file 2: Figure S2A). Meanwhile, we also detected the stemness-related genes expression [28] in A549-TR spheres and found that *Bmi1*, *KLF4*, *c-Myc*, *Nanog* and *Sox2* genes were indeed highly expressed (Fig. 2E). These findings proved that the acquired resistant cells had cancer stem cells properties. Additionally, since enriched differential genes showed metastasis correlation, epithelial mesenchymal transition (EMT) genes were investigated in resistant cells and the RT-qPCR results revealed elevated expression of *N-cadherin*, *Vimentin*, *Snail*, *Slug* and *ZEB1* genes (Fig. 2F). Given this, we may conclude that the resistant cells had the ability of cancer stem cells and metastasis.

### Inhibition of ErbB receptor reversed resistance of NSCLC

**Fig. 3:**
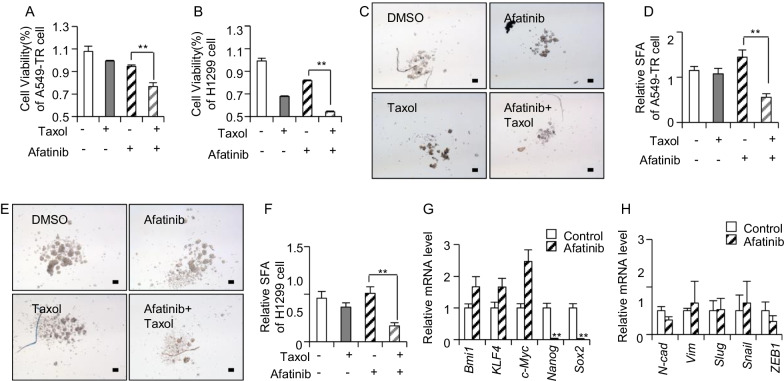
Inhibition of ErbB receptor reversed resistance of NSCLC. The cell viability of A549-TR cells (**A**) and H1299 cells (**B**) treated with 1 µM afatinib combined with 100 ng/mL taxol for 48 h, n = 3. **C**, **D** The representative images (**C**) and the statistics data (D) of A549-TR spheres treated with 1 µM afatinib combined with 100 ng/mL taxol, n = 3. **E**, **F** The representative images (**E**) and the statistics data (**F**) of H1299 spheres treated with 1 µM afatinib combined with 100 ng/mL taxol, n = 3. (**G**) RT-qPCR detection for the stemness-associated genes in A549-TR cells treated with 1 µM afatinib, n = 3. (**H**) RT-qPCR detection for EMT-related genes in A549-TR cells treated with 1 µM afatinib for 48 h, n = 3. Scale bars, 100 μm in black; TR, taxol resistance; *, *p* < 0.01; **, *p* < 0.01

GO analysis of 32 candidate resistance genes had revealed that receptor tyrosine kinase signaling were supposed to be activated in resistant cells. To further prove the involvement of RTK signaling pathways in drug resistance, we treated the resistant A549 cells with ErbB receptor inhibitor, afatinib [29], in combination with chemo-drugs to determine whether resistance of cancer cells to chemo-drug could be reversed. When A549-TR and H1299 cells were treated with 1 µM afatinib, it was observed that afatinib treatment suppressed the growth of A549-TR or H1299 cells slightly. However, combination of afatinib along with taxol had significantly inhibited viability of A549-TR or H1299 cells compared with taxol alone treatment (Fig. 3A and B). Similarly, concurrent treatment of afatinib also slightly suppressed the growth of A549-CR or H1299 cells alone, whereas a combination of afatinib together with cisplatin had remarkably repressed viability of A549-CR or H1299 cells (Additional file 3: Figure S3A and S3B). Therefore, it can be concluded that afatinib was capable of attenuating the resistance ability of cells, thus re-sensitizing these resistant cells to taxol and cisplatin drugs.

Moving on, it was still uncertain whether afatinib treatment could inhibit the sphere forming ability of A549-TR cells or H1299 cells. From the sphere forming assay, findings showed that 1 µM afatinib treatment alone did not suppress the sphere forming ability, but this was achieved when afatinib treatment was combined with taxol (Fig. 3C–F). After that, expression of stemness-associated genes in A549-TR cells was observed and it was seen that expression of Nanog and SOX2 genes were suppressed by afatinib (Fig. 3G). This result proved that afatinib could attenuate resistance via suppressing stemness of A549-TR cells. Since resistant cells are also correlated with metastasis ability, EMT-related genes’ expression in A549-TR cells treated with afatinib was investigated. However, the results showed that afatinib did not inhibit the mRNA level of EMT related genes (Fig. 3H). Overall, it can be concluded that afatinib attenuated resistance via inhibiting the stemness of cancer cells.

### EREG promotes chemoresistance of NSCLC

**Fig. 4:**
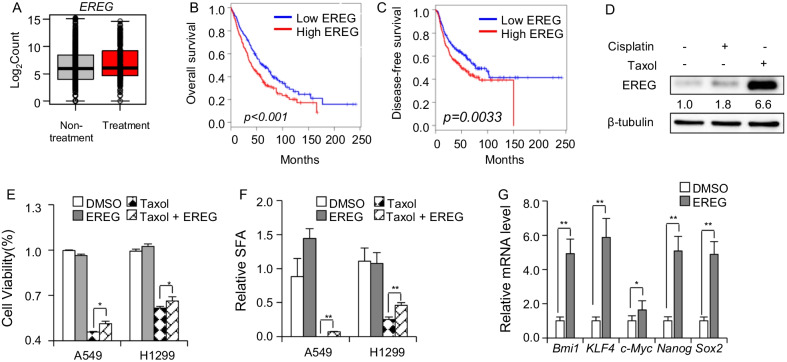
EREG promotes chemoresistance of NSCLC. **A** The EREG expression in non-treated lung cancer patients compared with treated lung cancer patients by RNA-Seq analysis. **B**, **C** Kaplan–Meier curves for overall survival (**B**) and disease-free survival (**C**) according to EREG expression levels in lung cancer patients. **D** WB detection of EREG protein level in cisplatin and taxol-treated A549 cells. **E** The cell viability of A549 cells and H1299 cells treated with 100 ng/mL EREG combined with 100 ng/mL taxol for 48 h, n = 3. **F** The statistics data of A549 and H1299 spheres treated with 100 ng/mL EREG plus with 100 ng/mL taxol. n = 3. **G** RT-qPCR detection for the stemness-associated genes in H1299 cells treated with 100 ng/mL EREG. n = 3. *, *p* < 0.01; **, *p* < 0.01

One of the validated resistance genes, EREG, is the ligand for ErbB receptor. However, EREG function in the chemoresistance regulation remains unclear. The RNA-Seq analysis revealed EREG level was higher in treated patients than untreated patients (Fig. 4A). Consistently, the overall survival time and disease-free survival time of EREG^high^ population were shorter than EREG^low^ population (Fig. 4B, C), which indicated highly expression of EREG promoted the progression of lung cancer and implied that EREG was correlated with resistance. To figure out whether EREG functions in chemoresistance, the EREG protein level was detected in A549 cells treated with cisplatin or taxol and the result showed that chemo-drugs treatment significantly elevated EREG level (Fig. 4D). This finding proposed that increased EREG level might cause chemoresistance of NSCLC. Subsequently, EREG cytokine was applied to treat the A549 and H1299 cells and the result revealed that the cell viability of EREG plus taxol-treated group was higher than the taxol-treated group (Fig. 4E). From sphere forming assay, it also revealed that the spheres of EREG plus taxol-treated group was significantly higher than the taxol-treated group (Fig. 4F). These findings indicated that EREG promoted chemoresistance of NSCLC. Interestingly, EREG treatment significantly increased the mRNA level of stemness-associated genes, Bmi1, KLF4, c-Myc, Nanog and Sox2 (Fig. 4G). Taken together, it concluded that EREG was able to promote chemoresistance of NSCLC via increasing the level of stemness-associated genes.

### Inhibition of ErbB receptor suppressed ERK signaling

**Fig. 5:**
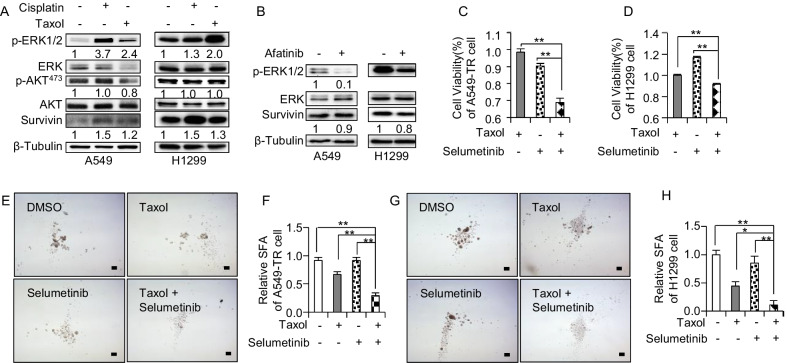
Inhibition of ErbB receptor suppressed ERK signaling. **A** WB detection for p-ERK1/2, p-AKT and Survivin in A549 cells and H1299 treated with cisplatin or taxol for 24 h. **B** WB detection for p-ERK1/2 and Survivin in A549-TR cells and H1299 cells treated with afatinib for 12 h. **C**, **D** The cell viability of A549 -TR cell (**C**) or H1299 cells (**D**) treated with 100 ng/mL taxol plus with 1 µM Selumetinib for 48 h, n = 3. **E**, **F** The representative images (**E**) and the statistics data (**F**) of A549-TR spheres treated with 100 ng/mL taxol plus with 1 µM Selumetinib, n = 3. **G**, **H** The representative images (**G**) and the statistics data (**H**) of H1299 spheres treated with 100 ng/mL taxol plus with 1 µM Selumetinib, n = 3. Scale bars, 100 μm in black; TR, taxol resistance; n.s., no significance; *, *p* < 0.05; **, *p* < 0.01

It was reported that ERK and AKT signaling was the downstream targets of receptor tyrosine kinase signaling [30–32]. Therefore, p-ERK1/2 and p-AKT expression in A549 and H1299 cells treated with cisplatin or taxol was analyzed. The results revealed that p-ERK1/2 was elevated significantly although p-AKT expression remained unchanged (Fig. 5A). This observation indicated that ERK signaling was primarily involved in drug resistance. Based on our findings so far, it was speculated that afatinib attenuated resistance via inhibition of ERK signaling. To verify this speculation, p-ERK1/2 level in A549-TR and H1299 cells treated with afatinib was tested and indeed, it was found that p-ERK1/2 expression had decreased significantly (Fig. 5B).

To further confirm that afatinib attenuated resistance through ERK signaling, p-EKR1/2 inhibitor (selumetinib) was used to treat A549-TR cells and the result showed that selumetinib had suppressed p-ERK1/2 (Additional file 4: Figure S4A). Along with this observation, selumetinib was also seen to enhance the sensitivity of A549-TR/CR cells to taxol and cisplatin (Fig. 5C and Additional file 4: S4B). We also tested the selumetinib effect on H1299 cells and findings obtained showed that selumetinib had assisted taxol and cisplatin to kill H1299 cells more effectively (Fig. 5D and Additional file 4: Figure S4C). Since afatinib treatment inhibited the stemness-associated genes expression and its combination with taxol had suppressed sphere forming ability, we therefore subsequently studied whether selumetinib could also suppress sphere forming ability when it was combined with taxol or cisplatin. The results proclaimed that selumetinib did not inhibit sphere forming ability when treated as a standalone drug, but combination of selumetinib and taxol or cisplatin was capable to effectively inhibit sphere forming activities in A549-TR and H1299 cells (Fig. 5E–H and Additional file 4: Figure S4D, S4E). In summary, EREG/ErbB played a role in drug resistance mediated by ERK signaling.

### Downregulation of EREG re-sensitized NSCLC to chemo-drugs through ERK signaling

**Fig. 6:**
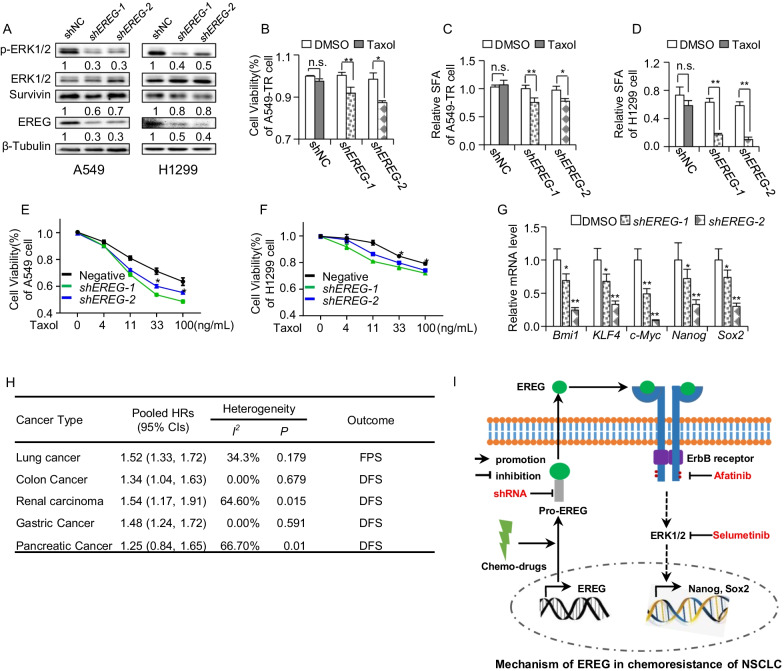
Downregulation of EREG re-sensitized NSCLC to chemo-drugs through ERK signaling. **A** WB detection for p-ERK1/2, Survivin and EREG in A549 and H12999 cells reconstituted with *shEREG*. **B** The cell viability of *shEREG* A549-TR cells treated with 100 ng/mL taxol for 48 h, n = 3 (**C**, **D**) The sphere forming of *shEREG* A549-TR (**C**) or *shEREG*H1299 cells (**D**) treated with 100 ng/mL taxol, n = 3. **E**, **F** The cell viability of *shEREG*-A549 cells (**E**) or *shEREG*-H1299 (**F**) treated with serial taxol for 48 h, n = 3. **G** The RT-qPCR detection of stemness-associated genes in A549-TR cells with *shEREG*, n = 3. **H** The HRs of positive resistant genes between highly expressed population and lowly expressed population in variant cancer. **I** Proposed schematic mechanism of EREG regulating stem-like properties in NSCLC via ERK pathway. HR, hazard ratio; CIs, confidence interval; TR, taxol resistance; n.s., no significance; *p < 0.05; **p < 0.01

EREG was knocked down in A549 and H1299 cells to determine whether inhibition of EREG could really reversed resistance. As shown in Fig. 6A, both *shEREG-1* and *shEREG-2* had effectively decreased the EREG protein level. Along with EREG knocked down, p-ERK1/2 and survivin expression was also seen to be decreased (Fig. 6A), which indicated that EREG activated ERK signaling. Furthermore, it was found that EREG knocked-down had rendered A549-TR/CR cells to be more sensitive to taxol and cisplatin drugs treatment (Fig. 6B and Additional file 5: Figure S5A). From the sphere forming assay, it was also found that *shEREG* had significantly increased sensitivity of the spheres derived from A549-TR and H1299 cells to taxol or cisplatin treatment (Fig. 6C, 6D and Additional file 5: Figure S5C). Similarly, A549-*shEREG* and H1299-*shEREG* cells were more sensitive to taxol or cisplatin treatment compared to parental cells (Fig. 6E, F and Additional file 5: Figure S5B). To further confirm that knocking down EREG affected the stemness, the mRNA level of stemness-associated genes was detected and it proclaimed that *shEREG* significantly decreased these stemness-associated genes level (Fig. 6G). Taken together, we concluded that knocking down EREG also reversed drug resistance by inhibition of stemness-associated genes through ERK signaling.

### Resistance gene signature was associated with disease-free survival in other cancers

To study whether this positive resistance gene signature, including *CYP24A1, DNAJC12, EREG, NPTX1, PAEP and TRPM8*, could also be a predictor signature for drug resistance in other cancer types, we analyzed the pooled HRs of disease-free survival time for these resistance genes in colon cancer, renal carcinoma, gastric cancer and pancreatic cancer. After that, a meta-analysis was conducted for pooled HRs of these genes. The pooled HRs of these genes in colon cancer was 1.34, which implied that these genes were a signature for drug resistance occurrence in colon cancer (Fig. 6H). Likewise, the pooled HRs of these genes in renal carcinoma and gastric cancer were 1.54 and 1.48, respectively, hence implying that these genes may also be signature for drug resistance occurrence in renal carcinoma and gastric cancer (Fig. 6H). On the other hand, the pooled HRs of pancreatic cancer was 1.25, where lower 95% CI is lesser than 1; therefore, it indicated that it was not significant for this resistance signature to act as a predictor in pancreatic cancer (Fig. 6H). From the meta-analysis, we concluded that this positive resistance gene signature was a predictor for drug resistance in lung cancer, colon cancer, renal carcinoma and gastric cancer.

## Discussion

Drug resistance tends to gradually occur after prolonged treatment with chemo-drug. Therefore, identification and targeting the acquired resistant NSCLC is imperative. In this study, the resistance signature was enriched by analyzing the comprehensive RNA-Seq data of lung cancer patients. Furthermore, this resistance signature was applied to other cancer types and its association with prognosis was validated. More importantly, we had proven that inhibition of EREG/ErbB signaling pathway played a key function in reversing resistance of NSCLC by suppressing stemness-associated genes, *Nanog* and *Sox2*, expression and inhibiting spheres forming ability, as mediated through ERK signaling (Fig. 6I). This finding shows that the inhibitor targeting the resistant genes or signaling at low concentration possibly removes the resistant cancer cells in combination with chemo-drugs.

EREG expression was reported to be associated with metastasis and shorter survival time in NSCLC [17], which was in agreement with our study, where lung cancer patients with high EREG mRNA level had a shorter survival time. Thus, EREG is already regarded as a therapeutic target in NSCLC [33]. A recent study revealed that EREG caused TKI resistance in NSCLC patients by suppressing apoptosis through EGFR/ERBB2 and AKT signaling pathways [34]. However, it was unsure whether EREG overexpression was associated with chemoresistance in NSCLC. In our study, we demonstrated that EREG promoted chemoresistance in NSCLC cells. Otherwise, EREG was demonstrated to be mainly expressed in macrophages through single-cell RNA sequencing, which then affected on NSCLC cells and caused its resistance [34]. However, our findings showed that the chemo-drugs led to increased EREG expression in NSCLC cells, which indicated that chemo-drug-induced acquired resistance might be attributed to EREG autocrine expression from tumor cells themselves, rather than a paracrine effect. Furthermore, EREG ligand binds to ErbB to activate AKT and ERK signaling [35]; however, we found that chemo-drugs increased EREG and ERK signaling without activation of AKT signaling, hence suggesting that ERK signaling is primarily responsible for chemoresistance rather than AKT. Importantly, EREG was correlated to the characteristics of cancer stem cells in esophageal cancer and colon cancer [16, 36]. But it remains unclear whether EREG also regulates cancer stem cells in NSCLC and promotes chemoresistance. In our study, the results revealed that EREG increased stemness-associated genes level to maintain the stemness of CSCs and to promote chemoresistance via ERK signaling.

Moreover, a study by *Keating* had revealed that afatinib not only inhibited the EGFR-WT NSCLC, but could also inhibit EGFR-mutant NSCLC [37]. Besides, more research showed that afatinib could effectively treat EGFR mutant NSCLC patients [38, 39]. This was further supported by one case report that revealed a longer survival period of the EGFR-mutant NSCLC patients who had received afatinib treatment followed by chemotherapy. This finding indicated that afatinib increased sensitivity of NSCLC to chemo-drug, which was in line with our observation where afatinib increased sensitivity of resistant A549 and parental cells to cisplatin or taxol. It also proved that targeting EGFR may enhance the therapeutic effect of chemo-drugs. Although previous study also screened the genes that played a role in resistance, however, the purpose of the inhibitor targeting the resistance-related genes was to kill the resistant cells [11]. The novelty of our finding is that 1 μM afatinib treatment alone did not inhibit the resistant cells, but only increased the sensitivity of resistant cells to chemo-drugs. This finding proposed that reversing resistant cancer cells to sensitive cancer cells may be a better way to solve the drug resistance issue, rather than identifying a new target in resistant cancer cells. Furthermore, we found that the resistant cells possessed both EMT and stemness characteristics that are involved in drug resistance [40, 41]. Nevertheless, the afatinib only downregulated the stemness-associated genes, but did not affect the EMT-associated genes expression. These results implied that inhibition of EREG/ErbB signaling did not suppress the proliferation of NSCLC, but instead reduced the stemness of NSCLC and hence, re-sensitizing the differentiated NSCLC to chemo-drug.

Regardless, there still exists some limitations in this study. Firstly, we found that the chemo-resistant NSCLC cells were also resistant to puromycin to some extent, which resulted in the purity of *shEREG*-infected cells that cannot reach 100% after puromycin selection. Besides, the positive resistance gene signature collectively could also be a drug resistance predictor for other cancers; however, not every gene was found to be positively correlated to DFS. Therefore, the signature has to be viewed as a whole to predicate resistance occurrence. Lastly, the mechanism of EREG/ERK regulation on stemness-associated genes remains unclear, which warrants further study.

## Conclusion

Our findings innovatively revealed a resistance signature of clinical NSCLC, which was enriched in receptor tyrosine kinase signaling and Vitamin D receptor signaling. Inhibition of ErbB receptor or knocking-down EREG suppressed ERK signaling, decreased expression of stemness-associated genes and finally affected the stemness of resistant cancer cells, which could re-sensitize the resistant cancer cells to chemo-drugs. This study discovered a resistance signature which could be a predictor for the chemoresistance of cancer. Additionally, inhibition of stemness is of clinical significance to solve resistance issue of NSCLC.

## Supplementary Information

**Additional file 1.** **Figure S1.** The expression of 32 resistant genes in lung cancer patients. (A) The counts for 13 resistance positively correlated genes. (B) The counts for 19 resistance negatively correlated genes. Y-axis, Log2Count of the RNA-Seq; grey column, non-treated patients; red column, treated patients.

**Additional file 2.** **Figure S2. **The resistant cells had stemness and EMT ability. The spheres assay of the A549-CR and A549-TR cells compared with A549 cells (2000 cells input, n=3). TR, taxol resistance; CR, cisplatin resistance; scale bars, 100 μm in black.

**Additional file 3.** **Figure S3. **Inhibition of ErbB receptor reversed resistance in NSCLC. (A) The cell viability of A549-CR cells treated with 1 µM afatinib combined with 4 μg/mL cisplatin for 48h, n=3. (B) The cell viability of H1299 cells treated with 1 µM afatinib combined with 4 μg/mL cisplatin for 48h, n=3. CR, cisplatin resistance; scale bars, 100 μm in black; *, *p*<0.05; **, *p*<0.01.

**Additional file 4.** **Figure S4**. Inhibition of ERK signaling reversed chemoresistance in NSCLC. (A) WB detection for p-ERK1/2 and Survivin in A549-TR treated with 1 µM selumetinib for 2h. (B)The cell viability of A549-CR cells treated with 1 µM selumetinib combined with 4 μg/mL cisplatin for 48h, n=3. (C) The cell viability of H1299 cells treated with 1 µM selumetinib combined with 4 μg/mL cisplatin for 48h, n=3. (D) The sphere forming ability of H1299 cells treated with 4 μg/mL cisplatin combined with 1 µM selumetinib , n=3. (E) The representative images for spheres of H1299 cells treated with 4 µg/mL cisplatin plus 1 µM selumetinib (2000 cells input, n=3). TR, taxol resistance; CR, cisplatin resistance; scale bars, 100 μm in black; n.s., no significance; *, *p*< 0.05; **, *p*<0.01.

**Additional file 5.** **Figure S5**. Downregulation of EREG re-sensitized NSCLC to chemo-drugs through ERK signaling. (A) The cell viability of *shEREG*-A549-CR cells treated with 4 μg/mL cisplatin for 48h, n=3. (B) The cell viability of *shEREG*-A549 cells treated with serial cisplatin for 48h, n=3. (C) The sphere forming of *shEREG*-H1299 cells treated with 4 μg/mL cisplatin, n=3. TR, taxol resistance; CR, cisplatin resistance; n.s., no significance; *, *p*< 0.05; **, *p*<0.01.

**Additional file 6.** **Table S1**. The upregulated genes in drug transport process.

**Additional file 7.** **Table S2**. The downregulated genes in collagen and apical part cell function.

**Additional file 8.** **Table S3**. The gene symbols of the GO analysis.

**Additional file 9.** **Table S4**. The HRs of the 32 candidate resistance genes in lung cancer.

**Additional file 10.** **Table S5**. The sequence of qPCR primers used in the study.

**Additional file 11.** **Table S6.** The list of antibodies used in the study.

## Data Availability

The datasets analyzed during the current study are available from the corresponding author on reasonable request.

## Abbreviations

NSCLC: Non-small cell lung cancer
TCGA: The cancer genome atlas
EREG: Epiregulin
HR: Hazard ratios
CI: Confidence intervals
CR: Cisplatin resistant
TR: Taxol resistant
EMT: Epithelial mesenchymal transition
RTK: Receptor tyrosine kinase
