# Supplementary material for: Epiregulin increases stemness-associated genes expression and promotes chemoresistance of non-small cell lung cancer via ERK signaling
Source: Stem Cell Res Ther. 2022 May 12;13:197. doi: 10.1186/s13287-022-02859-3 (PMC9102725; doi:10.1186/s13287-022-02859-3)
Supplement: Supplementary file 1 — Additional file 1. Figure S1. The expression of 32 resistant genes in lung cancer patients. (A) The counts for 13 resistance positively correlated genes. (B) The counts for 19 resistance negatively correlated genes. Y-axis, Log2Count of the RNA-Seq; grey column, non-treated patients; red column, treated patients. [file 13287_2022_2859_MOESM1_ESM.pdf]

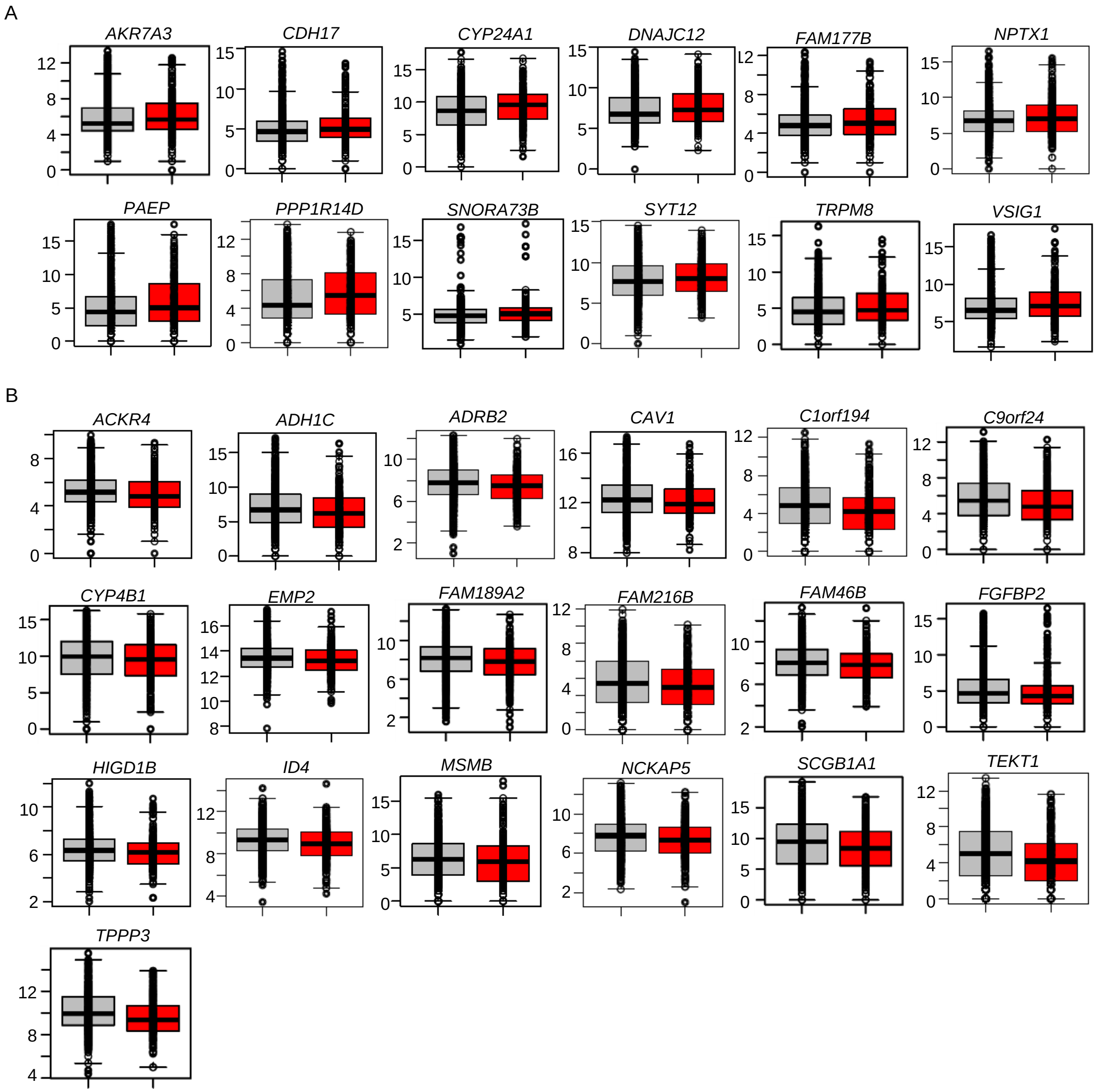

Figure S1. The expression of 32 resistant genes in lung cancer patients. (**A**) The counts for 13 resistance positively correlated genes. (**B**) The counts for 19 resistance negatively correlated genes. Y axis, Log_2Count of the RNA-Seq; grey column, non-treated patients; red column, treated patients.
