# Supplementary material for: Epiregulin increases stemness-associated genes expression and promotes chemoresistance of non-small cell lung cancer via ERK signaling
Source: Stem Cell Res Ther. 2022 May 12;13:197. doi: 10.1186/s13287-022-02859-3 (PMC9102725; doi:10.1186/s13287-022-02859-3)
Supplement: Supplementary file 2 — Additional file 2. Figure S2. The resistant cells had stemness and EMT ability. The spheres assay of the A549-CR and A549-TR cells compared with A549 cells (2000 cells input, n=3). TR, taxol resistance; CR, cisplatin resistance; scale bars, 100 μm in black. [file 13287_2022_2859_MOESM2_ESM.pdf]

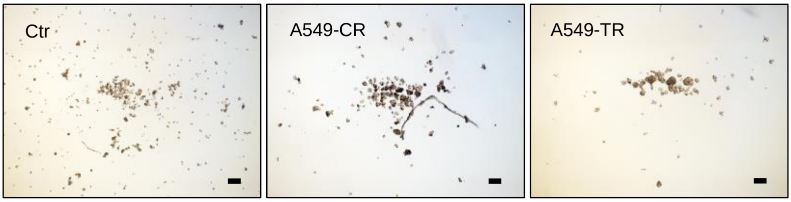

Figure S2. The resistant cells had stemness and EMT ability. The spheres assay of the A549-CR and A549-TR cells compared with A549 cells (2000 cells input, n=3). TR, taxol resistance; CR, cisplatin resistance; scale bars, 100 μm in black.
