# Supplementary material for: Epiregulin increases stemness-associated genes expression and promotes chemoresistance of non-small cell lung cancer via ERK signaling
Source: Stem Cell Res Ther. 2022 May 12;13:197. doi: 10.1186/s13287-022-02859-3 (PMC9102725; doi:10.1186/s13287-022-02859-3)
Supplement: Supplementary file 3 — Additional file 3. Figure S3. Inhibition of ErbB receptor reversed resistance in NSCLC. (A) The cell viability of A549-CR cells treated with 1 µM afatinib combined with 4 μg/mL cisplatin for 48h, n=3. (B) The cell viability of H1299 cells treated with 1 µM afatinib combined with 4 μg/mL cisplatin for 48h, n=3. CR, cisplatin resistance; scale bars, 100 μm in black; *, p<0.05; **, p<0.01. [file 13287_2022_2859_MOESM3_ESM.pdf]

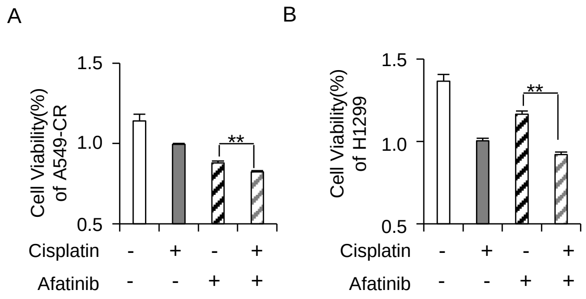

Figure S3. Inhibition of ErbB receptor reversed resistance in NSCLC. (**A**) The cell viability of A549-CR cells treated with 1 µM afatinib combined with 4 µg/mL cisplatin for 48h, n=3. (**B**) The cell viability of H1299 cells treated with 1 µM afatinib combined with 4 µg/mL cisplatin for 48h, n=3. CR, cisplatin resistance; *, p<0.05; **, p<0.01.
