# Supplementary material for: Epiregulin increases stemness-associated genes expression and promotes chemoresistance of non-small cell lung cancer via ERK signaling
Source: Stem Cell Res Ther. 2022 May 12;13:197. doi: 10.1186/s13287-022-02859-3 (PMC9102725; doi:10.1186/s13287-022-02859-3)
Supplement: Supplementary file 4 — Additional file 4. Figure S4. Inhibition of ERK signaling reversed chemoresistance in NSCLC. (A) WB detection for p-ERK1/2 and Survivin in A549-TR treated with 1 µM selumetinib for 2h. (B)The cell viability of A549-CR cells treated with 1 µM selumetinib combined with 4 μg/mL cisplatin for 48h, n=3. (C) The cell viability of H1299 cells treated with 1 µM selumetinib combined with 4 μg/mL cisplatin for 48h, n=3. (D) The sphere forming ability of H1299 cells treated with 4 μg/mL cisplatin combined with 1 µM selumetinib , n=3. (E) The representative images for spheres of H1299 cells treated with 4 µg/mL cisplatin plus 1 µM selumetinib (2000 cells input, n=3). TR, taxol resistance; CR, cisplatin resistance; scale bars, 100 μm in black; n.s., no significance; *, p< 0.05; **, p<0.01. [file 13287_2022_2859_MOESM4_ESM.pdf]

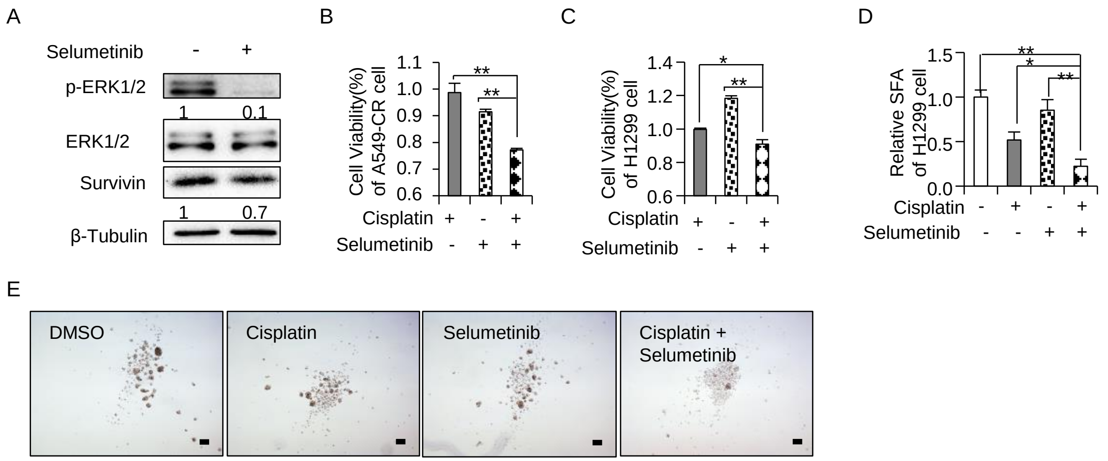

Figure S4. Inhibition of ERK signaling reversed chemoresistance in NSCLC. (**A**) WB detection for p-ERK1/2 and Survivin in A549-TR treated with 1 μM selumetinib for 2h. (**B**)The cell viability of A549-CR cells treated with 1 μM Selumetinib combined with 4 μg/mL cisplatin for 48h, n=3. (**C**) The cell viability of H1299 cells treated with 1 μM Selumetinib combined with 4 μg/mL cisplatin for 48h, n=3. (**D**) The sphere forming ability of H1299 cells treated with 4 μg/mL cisplatin combined with 1 μM Selumetinib , n=3. (**E**) The presentative images for spheres of H1299 cells treated with 4 μg/mL cisplatin plus 1 μM selumetinib (2000 cells input, n=3). TR, taxol resistance; CR, cisplatin resistance; scale bars, 100 μm in black; n.s., no significance; *, *p< 0.05*; **, p<0.01.
