# Supplementary material for: Epiregulin increases stemness-associated genes expression and promotes chemoresistance of non-small cell lung cancer via ERK signaling
Source: Stem Cell Res Ther. 2022 May 12;13:197. doi: 10.1186/s13287-022-02859-3 (PMC9102725; doi:10.1186/s13287-022-02859-3)
Supplement: Supplementary file 5 — Additional file 5. Figure S5. Downregulation of EREG re-sensitized NSCLC to chemo-drugs through ERK signaling. (A) The cell viability of shEREG-A549-CR cells treated with 4 μg/mL cisplatin for 48h, n=3. (B) The cell viability of shEREG-A549 cells treated with serial cisplatin for 48h, n=3. (C) The sphere forming of shEREG-H1299 cells treated with 4 μg/mL cisplatin, n=3. TR, taxol resistance; CR, cisplatin resistance; n.s., no significance; *, p< 0.05; **, p<0.01. [file 13287_2022_2859_MOESM5_ESM.pdf]

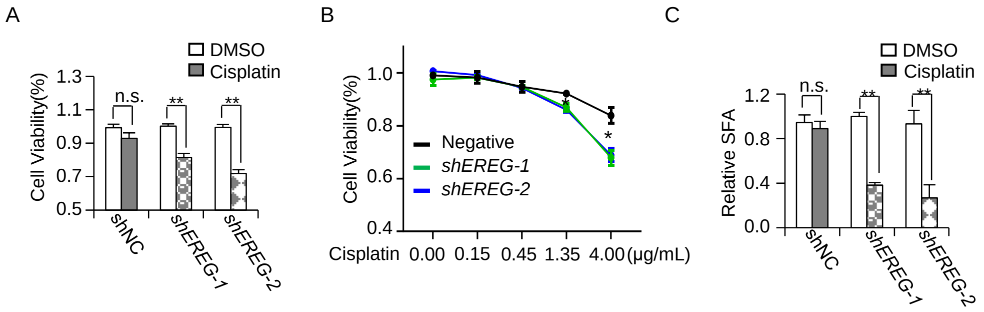

Figure S5. Downregulation of EREG re-sensitized NSCLC to chemo-drugs through ERK signaling. (**A**) The cell viability of *shEREG*-A549-CR cells treated with 4 μg/mL cisplatin for 48h, n=3. (**B**) The cell viability of *shEREG*-A549 cells treated with serial cisplatin for 48h, n=3. (**C**) The sphere forming of *shEREG*-H1299 cells treated with 4 μg/mL cisplatin, n=3. TR, taxol resistance; CR, cisplatin resistance; n.s., no significance; *, *p< 0.05*; **, p<0.01.
