# Supplementary material for: Epiregulin increases stemness-associated genes expression and promotes chemoresistance of non-small cell lung cancer via ERK signaling
Source: Stem Cell Res Ther. 2022 May 12;13:197. doi: 10.1186/s13287-022-02859-3 (PMC9102725; doi:10.1186/s13287-022-02859-3)
Supplement: Supplementary file 6 — Additional file 6. Table S1. The upregulated genes in drug transport process. [file 13287_2022_2859_MOESM6_ESM.docx]

**Table S1.** The upregulated genes in drug transport process

| SYMBOL | ENSEMBL | GENENAME |
| --- | --- | --- |
| SYT13 | ENSG00000019505 | synaptotagmin 13 |
| ABCC2 | ENSG00000023839 | ATP binding cassette subfamily C member 2 |
| AGT | ENSG00000135744 | angiotensinogen |
| AQP5 | ENSG00000161798 | aquaporin 5 |
| SLC1A7 | ENSG00000162383 | solute carrier family 1 member 7 |
| RAB3B | ENSG00000169213 | RAB3B, member RAS oncogene family |
| SYT12 | ENSG00000173227 | synaptotagmin 12 |
