# Supplementary material for: Epiregulin increases stemness-associated genes expression and promotes chemoresistance of non-small cell lung cancer via ERK signaling
Source: Stem Cell Res Ther. 2022 May 12;13:197. doi: 10.1186/s13287-022-02859-3 (PMC9102725; doi:10.1186/s13287-022-02859-3)
Supplement: Supplementary file 7 — Additional file 7. Table S2. The downregulated genes in collagen and apical part cell function. [file 13287_2022_2859_MOESM7_ESM.docx]

**Table S2**. The downregulated genes in collagen & apical part cell function

| SYMBOL | ENSEMBL | GENENAME |
| --- | --- | --- |
| MARCO | ENSG00000019169 | macrophage receptor with collagenous structure |
| SFTPA1 | ENSG00000122852 | surfactant protein A1 |
| SFTPD | ENSG00000133661 | surfactant protein D |
| FCN3 | ENSG00000142748 | ficolin 3 |
| SFTPA2 | ENSG00000185303 | surfactant protein A2 |
| COL6A6 | ENSG00000206384 | collagen type VI alpha 6 chain |
| ABCB1 | ENSG00000085563 | ATP binding cassette subfamily B member 1 |
| SLC1A1 | ENSG00000106688 | solute carrier family 1 member 1 |
| CLIC5 | ENSG00000112782 | chloride intracellular channel 5 |
| ECRG4 | ENSG00000119147 | ECRG4 augurin precursor |
| TEK | ENSG00000120156 | TEK receptor tyrosine kinase |
| CD36 | ENSG00000135218 | CD36 molecule |
| CA4 | ENSG00000167434 | carbonic anhydrase 4 |
| ADRB2 | ENSG00000169252 | adrenoceptor beta 2 |
| AGER | ENSG00000234729 | advanced glycosylation end-product specific receptor |
| EMP2 | ENSG00000213853 | epithelial membrane protein 2 |
| FXYD1 | ENSG00000266964 | FXYD domain containing ion transport regulator 1 |
| GPIHBP1 | ENSG00000277494 | glycosylphosphatidylinositol anchored high density lipoprotein binding protein 1 |
