# Supplementary material for: Epiregulin increases stemness-associated genes expression and promotes chemoresistance of non-small cell lung cancer via ERK signaling
Source: Stem Cell Res Ther. 2022 May 12;13:197. doi: 10.1186/s13287-022-02859-3 (PMC9102725; doi:10.1186/s13287-022-02859-3)
Supplement: Supplementary file 8 — Additional file 8. Table S3. The gene symbols of the GO analysis. [file 13287_2022_2859_MOESM8_ESM.docx]

**Table S3.** The gene symbols of the GO analysis.

| **Term** | **Description** | **Log*P*** | **Symbols** |
| --- | --- | --- | --- |
| GO:0007566 | embryo implantation | -4.59749 | EMP2,SCGB1A1,TPPP3 |
| R-HSA-211859 | Biological oxidations | -3.94273 | ADH1C,CYP4B1,CYP24A1,AKR7A3 |
| GO:0120162 | positive regulation of cold-induced thermogenesis | -3.7369 | ADRB2,CAV1,TRPM8,EREG,NPTX1,VSIG1,EMP2,NCKAP5 |
| WP2877 | Vitamin D Receptor Pathway | -2.92545 | ADRB2,**CYP24A1**,ID4 |
| R-HSA-9006934 | Signaling by Receptor Tyrosine Kinases | -2.60607 | CAV1,**EREG**,ID4,FGFBP2,ADH1C |
