# Supplementary material for: Epiregulin increases stemness-associated genes expression and promotes chemoresistance of non-small cell lung cancer via ERK signaling
Source: Stem Cell Res Ther. 2022 May 12;13:197. doi: 10.1186/s13287-022-02859-3 (PMC9102725; doi:10.1186/s13287-022-02859-3)
Supplement: Supplementary file 9 — Additional file 9. Table S4. The HRs of the 32 candidate resistance genes in lung cancer. [file 13287_2022_2859_MOESM9_ESM.docx]

**Table S4**: The HRs of the 32 candidate resistance genes.

| **Gene name** | **HR** | **Low 95% CI** | **Up 95% CI** |
| --- | --- | --- | --- |
| *ACKR4* | 0.60 | 0.49 | 0.73 |
| *HIGD1B* | 0.63 | 0.52 | 0.76 |
| *FAM189A2* | 0.74 | 0.59 | 0.93 |
| *FGFBP2* | 0.67 | 0.49 | 0.92 |
| *CYP4B1* | 0.46 | 0.33 | 0.64 |
| *SCGB1A1* | 0.71 | 0.59 | 0.86 |
| *TENT5B/FAM46B* | 0.68 | 0.52 | 0.89 |
| *TPPP3* | 0.68 | 0.55 | 0.83 |
| *C9orf24* | 0.67 | 0.51 | 0.89 |
| *TEKT1* | 0.60 | 0.43 | 0.84 |
| *ADRB2* | 0.52 | 0.41 | 0.67 |
| *ID4* | 0.55 | 0.43 | 0.7 |
| *NCKAP5* | 0.67 | 0.59 | 0.86 |
| *FAM216B* | 0.59 | 0.43 | 0.82 |
| *ADH1C* | 0.55 | 0.44 | 0.69 |
| *CAV1* | 0.46 | 0.36 | 0.59 |
| *C1orf194* | 0.76 | 0.56 | 1.03 |
| *EMP2* | 0.58 | 0.47 | 0.7 |
| *SNORA73B* | 1 | 0.9998 | 1.0002 |
| *MSMB* | 1.54 | 1.24 | 1.92 |
| *FAM177B* | 1.6 | 1.1427 | 2.2402 |
| *VSIG1* | 1.36 | 1.02 | 1.8 |
| *NPTX1* | 1.4 | 1.006 | 1.9483 |
| *CYP24A1* | 1.28 | 1.02 | 1.6 |
| *CDH17* | 1.28 | 1.04 | 1.57 |
| *DNAJC12* | 1.54 | 1.18 | 2.02 |
| *PAEP/GD* | 1.94 | 1.56 | 2.41 |
| *EREG* | 1.45 | 1.18 | 1.79 |
| *TRPM8* | 1.78 | 1.31 | 2.42 |
| *AKR7A3* | 1.56 | 1.29 | 1.89 |
| *PPP1R14D* | 2.08 | 1.65 | 2.62 |
| *SYT12* | 1.69 | 1.29 | 2.22 |

Note: HR, Hazard Ratio; CI, Confidence Interval.
