# Supplementary material for: Epiregulin increases stemness-associated genes expression and promotes chemoresistance of non-small cell lung cancer via ERK signaling
Source: Stem Cell Res Ther. 2022 May 12;13:197. doi: 10.1186/s13287-022-02859-3 (PMC9102725; doi:10.1186/s13287-022-02859-3)
Supplement: Supplementary file 10 — Additional file 10. Table S5. The sequence of qPCR primers used in the study. [file 13287_2022_2859_MOESM10_ESM.docx]

**Table S5**: The sequence of qPCR primers

| **Gene** | **primer Sequence** |
| --- | --- |
| *CYP24A1* | F: 5'- TTGGCTCTTTGTTGGATTGTC -3' |
|  | R: 5'- TGAACCCTGTAGAATGCCTTG -3' |
| *DNAJC12* | F: 5'- CCCAAGCCCCTAGAGAAGTC -3' |
|  | R: 5'- GTTCTGAGGGAGCATCCTTG -3' |
| *EREG* | F: 5'- ATGGCTATTGTTTGCATGGAC -3' |
|  | R: 5'- CTCTGGATCCCCTGAGGTAAC -3' |
| *NPTX1* | F: 5'- TGGAGATCCTCATCAATGACA -3' |
|  | R: 5'- AGATCTCGATGTGGGATTCAG -3' |
| *PAEP* | F: 5'- GAGCATGATGTGCCAGTACCT -3' |
|  | R: 5'- ATGACCACGTCATCGTCTTCT -3' |
| *TRPM8* | F: 5'- CAAGTGTTGCTGCAAGGAGA -3' |
|  | R: 5'- GCCTCATTTCCTCTGAGGTG -3' |
| *EMP2* | F: 5'- ATGTTCCAGGCACTGTGTGA -3' |
|  | R: 5'- TCCTCCATGAGCTTGGGTAG -3' |
| *HIGD1B* | F: 5'- ATAGGCTTAGGAGGCTGCTTG -3' |
|  | R: 5'- TCCTCTTGACGTAATCGCTGT -3' |
| *ADH1C* | F: 5'- CCAAACTTGTGGCTGAC -3' |
|  | R: 5'- TTTAAACATTTGCTTGAC -3' |
| *GAPDH* | F: 5'- AGCCACATCGCTCAGACAC -3' |
|  | R: 5'- GAGGCATTGCTGATGATCTTG -3' |
| *N-Cadherin* | F: 5'- TTATCCTTGTGCTGATGTTTGTG -3' |
|  | R: 5'- TAACAGGGAGTCATATGGTGGAG -3' |
| *VIM* | F: 5'- TCTGGATTCACTCCCTCTGG -3' |
|  | R: 5'- GCAGAAAGGCACTTGAAAGC -3' |
| *Slug* | F: 5'- ATATTCGGACCCACACATTACC -3' |
|  | R: 5'- TTGCGTCACTCAGTGTGCTAC -3' |
| *Snail1* | F: 5'- CCTCCCTGTCAGATGAGGAC -3' |
|  | R: 5'- CCAGGCTGAGGTATTCCTTG -3' |
| *ZEB1* | F: 5'- AACCACCCTTGAAAGTGATCC -3' |
|  | R: 5'- CTCTTCCTGCTCTGTGCTGTC -3' |
| *BMI1* | F: 5'- AGATACTTACGATGCCCAGCAG -3' |
|  | R: 5'- CAGTCCATCTCTCTGGTGACTG -3' |
| *MYC* | F: 5'- TCTGAGGAGGAACAAGAAGATG -3' |
|  | R: 5'- GCGTAGTTGTGCTGATGTGTG -3' |
| *Nanog* | F: 5'- ATAGCAATGGTGTGACGCAG -3' |
|  | R: 5'- CCAGGTTGAATTGTTCCAGGTC -3' |
| *KLF4* | F: 5'- CATGCCAGAGGAGCCCAAG -3' |
|  | R: 5'- ATTTCCATCCACAGCCGTCC -3' |
| *Sox2* | F: 5'- GGGAAATGGGAGGGGTGC -3' |
|  | R: 5'- TGCGTGAGTGTGGATGGG -3' |

Note: F, forward primer; R, reverse primer
