# Supplementary material for: Epiregulin increases stemness-associated genes expression and promotes chemoresistance of non-small cell lung cancer via ERK signaling
Source: Stem Cell Res Ther. 2022 May 12;13:197. doi: 10.1186/s13287-022-02859-3 (PMC9102725; doi:10.1186/s13287-022-02859-3)
Supplement: Supplementary file 11 — Additional file 11. Table S6. The list of antibodies used in the study. [file 13287_2022_2859_MOESM11_ESM.docx]

**Table S6.** The list of antibodies used in the study

| **Antibody** | **Vendor^1^** | **Clone / Cat. no.** | **Species^2^** | **WB applications^3^** |
| --- | --- | --- | --- | --- |
| AKT | CST | C67E7 | Rb | 1:2000 |
| p-AKT (473) | CST | D9E | Rb | 1:1000 |
| β-Tubulin | EB | 6C4 | M | 1:5000 |
| ERK1/2 | CST | 137F5 | Rb | 1:2000 |
| p-ERK1/2 | CST | D13.14.4E | Rb | 1:2000 |
| Survivin | CST | 71G4B7 | Rb | 1:1000 |
| EREG | CST | D4O5I | Rb | 1:1000 |
| 1. CST, Cell Signaling Technology; EB, Engibody Biotechnology (Beijing, China); | | | | |
| 2. M, mouse; Rb , rabbit; Rt , | | |  |  |
